# The Law Relating to Infectious Diseases.—V

**Published:** 1902-09-20

**Authors:** J. E. R. Stephens

**Affiliations:** Barrister-at-law, Author of "Digest of Public Health Cases," etc. etc.


### The Institutional Workshop.

### THE LAW RELATING TO INFECTIOUS DISEASES.—V.

By J. E. R. Stephens, Barrister-at-law, Author of "Digest of Public Health Cases," etc. etc.

### Epidemic Diseases (Prevention of).

Sect. 134 of the Public Health Act, 1875 gives power to the Local Government Board to make regulations for prevention of diseases. It enacts that whenever any part of England appears to be threatened with, or is affected by, any formidable epidemic, endemic, or infectious disease, the Local Government Board may make, and from time to time alter, and revoke regulations for all or any of the following purposes; namely, (1) for the speedy interment of the dead; and (2) for house-to-house visitation; and (3) for the provision of medical aid and accommodation, for the promotion of cleansing, ventilation, and disinfection, and for guarding against the spread of disease; and may by order declare all or any of the regulations so made to be in force within the whole or any part or parts of the district of any local authority, and to apply to any vessels whether on inland waters or on arms or parts of the sea within the jurisdiction of the Lord High Admiral of the United Kingdom; and may by any subsequent order, abridge or extend such period.

Under section 130 the Local Government Board may from time to time make, alter, and revoke such regulations as to the said board may seem fit, with a view to the treatment of persons affected with cholera, or any other epidemic, endemic, or infectious disease, and preventing the spread of cholera and such other diseases, as well on the seas, rivers, and waters of the United Kingdom, and on the high seas within three miles of the coasts thereof, as on land, and may declare by what authority or authorities such regulations shall be enforced and executed. Regulations so made shall be published in the London *Gazette*, and such publication shall be for all purposes conclusive evidence of such regulations.

The above provisions of the Public Health Act, 1875, are extended to London by section 113 of the Public Health (London) Act, 1891. The Public Health Act, 1896, imposes a penalty of £100 for breach of the regulations made under the above sections, and a further penalty of £50 a day in the case of a continuing offence. Regulations for the removal of infected persons brought into the district by ships may be made by the local authority under section 125.

Various orders prohibiting the importation of rags from places infected with cholera have been issued by the Local Government Board. By-laws for preventing the spread of infectious diseases from tents, vans, sheds, and similar structures used for human habitation may be made by sanitary authorities under the Housing of the Working Classes Act, 1885.

By sect. 136 of the Act of 1875, the local authority of any district within which or part of which regulations so issued by the Local Government Board are declared to be in force, shall superintend and see to the execution thereof, and shall appoint and pay such medical or other officers or persons, and do and provide such acts, matters, and things as may be necessary for mitigating any such disease, or for superintending or aiding in the execution of such regulations, or for executing the same, as the case may require. Moreover, the local authority may from time to time direct any prosecution or legal proceedings for or in respect of the wilful violation or neglect of any such regulation.

Under sect. 137 the local authority and their officers shall have power of entry on any premises or vessel for the purpose of executing or superintending the execution of any regulations so issued by the Local Government Board.

Whenever, in compliance with any regulation so issued by the Local Government Board, any Poor Law medical officer performs any medical service on board any vessel he shall be entitled to charge extra for such service at the general rate of his allowance for services for the union or place for which he is appointed; and such charges shall be payable by the captain of such vessel on behalf of the owners thereof, together with any reasonable expenses for the treatment of the sick. Where such services are rendered by any medical practitioner who is not a Poor Law medical officer, he shall be entitled to charges for any service rendered on board, with extra remuneration on account of distance, at the same rate as those which he is in the habit of receiving from private patients of the class of those attended and treated on shipboard, to be paid as aforesaid. In case of dispute in respect to such charges, such dispute may, where the charges do not exceed £20, be determined by a court of summary jurisdiction; and such court shall determine summarily the amount which is reasonable, according to the accustomed rate of charge within the place where the dispute arises for attendance on patients of the like class as those in respect of whom the charge is made (sect. 138).

### Exposure of Infected Persons.

Sect. 126 of the Public Health Act, 1875, enacts that any person who (1) while suffering from any dangerons infectious disorder wilfully exposes himself without proper precautions against spreading the said disorder on any street, public place, shop, inn or public conveyance, or enters any public conveyance without previously notifying to the owner, conductor or driver thereof that he is so suffering; or (2) being in charge of any person so suffering, so exposes such sufferer; or (3) gives, lends, sells, transmits or exposes, without previous disinfection, any bedding, clothing, rags or other things which have been exposed to infection from any such disorder, shall be liable to a penalty not exceeding £5; and a person who, while suffering from any such disorder, enters any public conveyance without previously notifying to the owner or driver that he is so suffering, shall in addition be ordered by the Court to pay such owner and driver the amount of any loss and expense they may incur in carrying into effect the provisions of this Act with respect to disinfection of the conveyance. Provided that no proceedings under this section shall be taken against persons transmitting with proper precautions any bedding, clothing, rags, or other things for the purpose of having the same disinfected. And by section 127 every owner or driver of a public conveyance shall immediately provide for the disinfection of such conveyance after it has to his knowledge conveyed any person suffering from a dangerous infectious disorder; and if he fails to do so he shall be liable to a penalty not exceeding £5, but no such owner or driver shall be required to convey any person so suffering until he has been paid a sum sufficient to cover any loss or expense incurred by him in carrying into effect the provisions of this section.

In places where the Infectious Diseases (Prevention) Act, 1890, has been adopted, it is unlawful to keep the body of a person who has died of infectious disease in a dwelling place, sleeping place, or workroom for more than 48 hours, or to use a public conveyance for carrying any such body without notifying the fact to the owner of the conveyance. The same Act makes provisions for the disinfection of conveyances used to carry dead bodies, and for the compulsory disinfection of infected bedding, clothing, and other materials.

It was held in *Rex* v. *Vantandillo* (4 M. & S. 73) to be an indictable offence for a person "to unlawfully and injuriously carry a person suffering from an infectious disease along a public highway in which persons were passing, and near to the habitation of the King's subjects." So also if a person cause others infected with a contagious disease to be carried along a public street; as where the defendant caused patients inoculated with the small-pox to be brought to his surgery whilst infected with the disease. (*Rex* v. *Burnett* 4 M. & S. 272). In *Reg.* v. *Hanson* (Dears C. C. 24) a person was indicted for bringing a horse diseased with glanders into a public place, to the danger of the Queen's subjects. A medical man in practice in Tunbridge Wells sent a patient who was suffering from scarlet fever to the fever hospital there with a certificate, directing him to walk in the middle of the road, and not to talk to anyone, but, in consequence of an alleged informality in the certificate, the patient was refused admission; whereupon the medical man walked with him through the streets of the town to the residence of the chairman of the local board, from whom, after some delay, he obtained an order for the man's admission to the hospital. He then returned with the patient to the police-station to procure the ambulance to convey him thither. Upon an information against the medical man for an alleged infringement of the statute, the justices were of opinion that it was not proved before them that the medical man "had charge of" the patient, that he had not wilfully exposed the patient in any street or public place "without proper precaution," and that he had made the best use of the means at his disposal to prevent the spread of the fever; and they refused to convict him. It was held that the justices were right (*Tunbridge Wells Local Board* v. *Bisshopp*, 2 C.P.D., 187). In another case, that of the *Managers of the Metropolitan Asylum District* v. *Hill* (45 J.P., 664), Lord Blackburn said: "Where those who have the custody of the person sick of an infectious disorder have not the means of isolating him from the other inmates, which is very commonly the case with the poor, and consequently those other inmates and their neighbours are exposed to the risk of infection, I think that the inability to isolate him would form a sufficient excuse to be a defence to any indictment; and I think, also, though I am not aware of any authority on the subject, that the neighbours could not maintain any action for the damage which they would in such a case sustain from the proximity of the infected person, it being a necessary incident to the use of property for habitations in town that contagious sickness may befall their neighbours. If those who have the charge of the infected person have the means of isolating him on the spot, they certainly do well to use them, and if it cannot be done on the spot and they can, either by their own means or by the aid of charitable persons who have erected a hospital, find a place where he can be isolated so as to avoid the risk of infection, they will do well to use these means. I do not mean to express any opinion as to whether, at common law, they would or would not be responsible for not doing so, but there is no authority, and I think no principle for saying that they are justified in removing him to a place where the neighbours would be exposed to contagion, though it may be that those neighbours would be fewer in number than the neighbours of the spot where the infection broke out; nor for saying that if that was done, and the contagion was such as to amount to a real nuisance, those neighbours might not maintain an action, and obtain an injunction to protect themselves against the importation of foreign infection."

